# How Foot Tracking Matters: The Impact of an Animated Self-Avatar on Interaction, Embodiment and Presence in Shared Virtual Environments

**DOI:** 10.3389/frobt.2019.00104

**Published:** 2019-10-30

**Authors:** Ye Pan, Anthony Steed

**Affiliations:** Virtual Environments and Computer Graphics Group, Department of Computer Science, University College London, London, United Kingdom

**Keywords:** virtual reality, SVE, self-avatar, foot tracking, interaction

## Abstract

The use of a self-avatar representation in head-mounted displays has been shown to have important effects on user behavior. However, relatively few studies focus on feet and legs. We implemented a shared virtual reality for consumer virtual reality systems where each user could be represented by a gender-matched self-avatar controlled by multiple trackers. The self-avatar allowed users to see their feet, legs and part of their torso when they looked down. We implemented an experiment where participants worked together to solve jigsaw puzzles. Participants experienced either no-avatar, a self-avatar with floating feet, or a self-avatar with tracked feet, in a between-subjects manipulation. First, we found that participants could solve the puzzle more quickly with self-avatars than without self-avatars; but there was no significant difference between the latter two conditions, solely on task completion time. Second, we found participants with tracked feet placed their feet statistically significantly closer to obstacles than participants with floating feet, whereas participants who did not have a self-avatar usually ignored obstacles. Our post-experience questionnaire results confirmed that the use of a self-avatar has important effects on presence and interaction. Together the results show that although the impact of animated legs might be subtle, it does change how users behave around obstacles. This could have important implications for the design of virtual spaces for applications such as training or behavioral analysis.

## 1. Introduction

With the launch of consumer head-mounted displays. shared virtual environments (SVE) have rapidly increased in popularity as a form of remote collaboration. One side-effect of wearing a head-mounted display is that users cannot see their own bodies. Thus, it is common for such systems to include a *self-avatar*, a virtual representation of a body that is depicted from the first-person perspective of the user. However, most current applications don't include a complete animated self-avatar, partly due to the limited tracking. In particular, while the head and hands are usually tracked, the body and feet are not commonly captured. Some applications only display a partial representation of the user, such as the controllers or models of the hands (e.g., Toybox demo for oculus touch, Oculus, 2017); while others show a static self-avatar (e.g., AltspaceVR, AltspaceVR, 2017). However, the utility of a full body self-avatar has been shown to have a positive benefit to the sense of presence, interaction tasks and perceptual judgments (see section 2.2).

We ask whether the presence of a complete self-avatar can aid in performance of tasks in an SVE and specifically whether foot tracking can influence users' behavior.

We developed a virtual reality system where several users can meet and interact in a shared virtual environment. Each participant was represented by a jointed self-avatar. Each user's head and hands were tracked via the HTC Vive's tracking system. Strapping one HTC Vive tracker to each user's waist, provided more accurate position information for the user's body (e.g., the user would be able to peek around corners, as their body position is no longer based on the HMD's position and rotation). Strapping a tracker to each of the user's feet allowed the user to look or reach down and see movement of their legs or feet (e.g., be able to step on things or play football). The full body movements of each user were transmitted through a server such that everyone can see each other's avatar and feel their presence in the virtual world.

We designed an experiment around a highly collaborative jigsaw puzzle task to be undertaken by a pair of users. We chose this hand-eye coordination task so that we could look at the impact of foot tracking on participants' unintentional actions or behavior during collaboration. Jigsaw pieces were spread over an office environment. Participants had to assemble the jigsaw on a central table. The office environment was quite compact and cluttered. We expected that participants might avoid stepping into objects when retrieving and placing the jigsaw pieces. We also expected that the use of a self-avatar might affect this behavior, and might affect other measures of presence, engagement and collaboration. We thus had three conditions. One third of dyads experienced the *floating feet* condition, where their avatar's feet and legs floated below their torso, and were thus asynchronous to their own foot movement. In one third of the dyads, both self-avatars featured *tracked feet* that they could control accurately and synchronously through foot movement. In the final third of the dyads, users experienced *no self-avatar*, and were thus represented only by representations of the controllers.

We found that participants completed the task more quickly with a self-avatar than they did without a self-avatar; but no significant effect was found in between tracked feet and floating feet in terms of task completion time. We then looked at the paths of participants while solving puzzles, including collisions with the obstacles and proximity to the obstacles. Statistically significant results were found: participants tended to deviate farther around obstacles in the floating feet condition than the tracked feet condition; and participants exhibited more collisions with obstacles without a self-avatar than with self-avatar. The post-experience questionnaire results further confirmed that the self-avatar provides a dominant effect in improving sense of ownership, agency and presence. There is a small additional effect of having foot tracking. Furthermore, observational results revealed that a self-avatar could convey obvious cues about the user's location and this non-verbal information aided collaboration. For example, participants with floating feet felt that it was hard to control their virtual feet, and thus detours around an obstacle might have been initiated earlier.

## 2. Related Work

### 2.1. SVE

The use of SVEs as a means of collaboration dates back at least to VPL's seminal reality built for two system from the late 1980s. In this system two users could see each in the virtual world as avatars. In the 1990s and 2000s many demonstrations of larger scale collaborative systems were built (see technical review in Steed and Oliveira, 2009). In those systems researchers started to notice the pronounced effect on social interaction that having an avatar had (e.g., see Maister et al., 2015 and the review in Schroeder, 2012). At the time though, virtual environment systems were relatively crude, and the underlying theory of embodiment was not developed and understood in a HCI content (Heldal et al., 2005; Otto et al., 2006; Yee et al., 2007). Recent work has significantly expanded our understanding of the function of avatars, see below. SVE technologies have continued to be used, mostly in gaming contexts with a few social applications such as SecondLife. However, these systems used desktop or games console-style interfaces.

In the past 5 years, consumer virtual reality systems have become widely available. Several lightweight, easily configurable SVE systems have been developed. For example, MuVR (Thomas et al., 2014) used an Oculus Rift HMD and a Razer Hydra tracking system to create multiuser virtual reality platform. MetaSpace, Sra and Schmandt (2015) included a self-avatar by tracking each user's body with a Kinect device.

Inspired by these recent systems, we developed an SVE system that provided a complete self-avatar for each user including animated legs and feet. We investigated how the mapping of a participant's movements onto their avatar affects their behavior in an SVE. We manipulated the tracking level (i.e., no tracking, tracking only the positions and orientation of the head and hands, and full body tracking).

### 2.2. Self-Avatar

The use of self-avatars that are visual embodiment of users, their interaction with the world, and sensation of the surrounding virtual environments have profound impacts on the user experience (e.g., Slater et al., 1995). Slater et al. (2009) have found that with a self-avatar a user has a perception that a virtual body is their own, resulting in so called “body-ownership illusions.” Yuan and Steed (2010) demonstrated how a user can begin to associate a virtual limb with their own body by participating in an interactive task via a HMD VR system. Researchers have furthermore shown how body ownership affects interaction style and task performance in virtual environments (see Steptoe et al., 2013; Spanlang et al., 2014; Argelaguet et al., 2016; Jung and Hughes, 2016; Steed et al., 2016; Feuchtner and Müeller, 2017; Schwind et al., 2017). The practical impacts of self-avatars have been extensively investigated as well (Ries et al., 2008; Bodenheimer et al., 2017). For example, Mohler et al. (2010) showed that an animated self-avatar was superior to that of a static one when users are tasked with distance estimation. More recently, Murphy (2017) observed that users may experience a sense of ownership and/or agency over their virtual actions even in the absence of visible avatar body parts. Kondo et al. (2018) also confirmed that visual hands and feet were sufficient to induce illusory body ownership, and this effect was as strong as using a whole-body avatar.

Self-avatars in an SVE have additional functions, including determining position, identification, visualization of focus of attention and recognition of gesture and actions (Benford et al., 1995; Bowers et al., 1996; Schultze, 2010; Roth et al., 2016, 2017). Dodds et al. (2011) have shown that the use of self-avatars provides a more effective communication medium when talking to another person in a multi-user VR system. McManus et al. (2011) found that users performed tasks more accurately and quickly when they were paired with a self-avatar.

Various papers have explored the impact of having higher visual fidelity or tracking accuracy of users including eye tracking, facial expression and finger tracking. Higher fidelity or accuracy can convey additional subtleties of human nonverbal communication, increasing the perceived authenticity of interactions in a virtual world (see Steptoe et al., 2008; Hodgins et al., 2010; Bodenheimer and Fu, 2015; Young et al., 2015). On the other hand, the use of self-avatars can generate issues that stem from the perceived “uncanny valley” when users begin to doubt the authenticity of virtual characters due to divergences from realistic human behavior (see Raij et al., 2007; McDonnell et al., 2012; Piwek et al., 2014; Kätsyri et al., 2015). Hussain et al. (2011) have found that the avatar's appearance has an impact of the credibility of the information they provide—observing that professional appearances in avatar designs correlate highly with perceived credibility by the users interacting with them. McDonnell et al. (2012) have shown that small differences in rendering details can influence perception of CG characters. Steptoe et al. (2010) found that realistic eye motion in avatars added depth to communication between users—enabling participants to detect the differences between truth and deception in their interactions. Hyde et al. (2015) found that simply adjusting the expressiveness of interactive animated avatars begins to change the measure of user's social judgments and their willingness to collaborate with animated avatars.

In this study we build on this past research. We look at how the self-avatar alters users' behavior and the collaboration outcomes in an SVE. We specifically address the question of how user behaviors change when foot tracking is enabled.

## 3. System Design

### 3.1. Technical Setup

The experiments were performed in two laboratory settings, each with dimensions 4m × 6m, in the same building. The two locations were networked such that while physically separated, the users were collaborating in the same virtual environment.

Each participant's virtual environment ran on an isolated computer application running on a Windows 8.1 workstation with an Intel Core i7 processor, 8 GB ram and a GeForce TitanX graphics card. Each user was provided with an HTC Vive headset to view the VR world with hand controllers and 3 Vive trackers to track limb movement. The virtual environment was created using Unity 5.6.2f and written in C#, with scenes rendered at 90 Hz. Extension cables were utilized for both audio and video transmission to ensure unobstructed movements by participants.

### 3.2. Scene, Flowers and Jigsaw Puzzle Pieces

**Figure 1:**
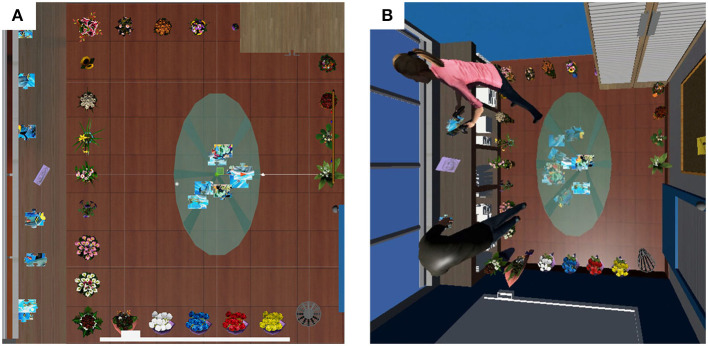
The virtual office environment. **(A)** Plan view. **(B)** A 3rd person view of the avatars of the two participants in the virtual environment solving a jigsaw puzzle. Participants with tracked feet tended to avoid collision with the flowers on the floor on their way to pick up the puzzle pieces from the shelves.

The background scene was a model of an office interior, including an oval glass conference table, a whiteboard, a waste paper bin, shelving, etc. See Figure 1 for a plan view of the environment. We modeled the office at the size of 3.6 × 3.6 m to ensure that the movement of the participants within the VE fit inside the boundaries of the their physical laboratory and their tracking space.

The jigsaw puzzle pieces were created using Puzzle Maker (2017). We cut the puzzle image into 16 pieces. The size of image was 40 × 60 cm, which was necessary to ensure that the pieces could be easily visible and manipulable in the HMD. Half of the pieces were scattered on the oval glass conference table (within 30 cm from the center of table), while others were placed on the shelving (within 10 cm from the long axis of the shelf, and with the points 50 cm apart from one another).

We placed flowers around the edge of the users' walking space, 20 cm apart from one another. The average size of the flowers was 30 cm tall with a radius of 10 cm.

### 3.3. Avatars

**Figure 2:**
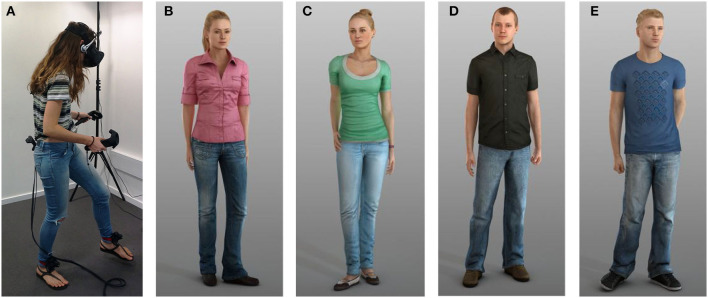
A suited participant with the Vive HMD, trackers, and controllers, and the self- avatar models used in the experiment. **(A)** User **(B)** Female model 1 **(C)** Female model 2 **(D)** Male model 1 **(E)** Male model 2.

Some participants had a randomly assigned gender-matched self-avatar (see Figure 2). We provided two male and two female avatars, taken from the Rocketbox Complete Characters HD set, in generic clothing. We needed two distinguishable self-avatars of each gender in the SVE so that pairs of users of the same gender had distinct avatars. We used each participant's height information to adjust and scale the height of the avatar.

We attached two Vive trackers to the participant's feet and one to their lower back (see Figure 2). The user wore the Vive HMD and held the two Vive controllers. This gave six points (two foot, pelvis, head and two hands) of tracking to animate the self-avatar. We then used the VR IK solver from the RootMotion (2017) Final IK plugin to map participant's movements in real world space to the self-avatar's movements.

### 3.4. Manipulation Technique

We implemented an object interaction system by using the Virtual Reality Toolkit (VRTK) (VRTK - Virtual Reality Toolkit, 2017). In the Unity scene, each puzzle piece required a collider to activate the trigger and a rigidbody to pick it up and move it around the game world. VRTK provided a script to attach to the controller to enable touch and grabbing. If a puzzle piece was touched then pressing the trigger on the controller will grab and snap the object to the controller. The puzzle piece was released when the trigger was released. Additionally, the Swap Controller Grab Action script from the VRTK provided a mechanism to allow puzzle pieces to be swapped between controllers.

### 3.5. Networking

**Figure 3:**
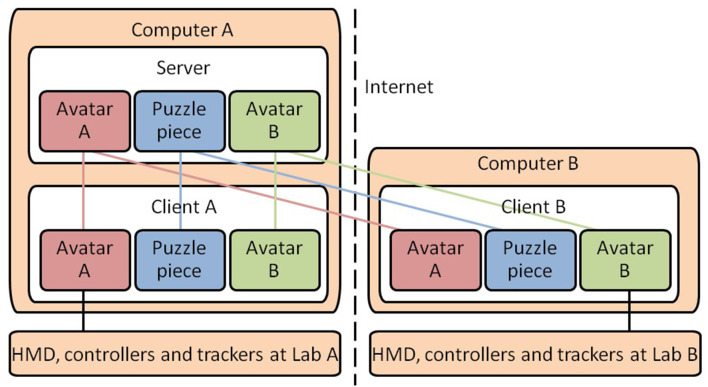
The networking diagram for our SVE system.

To ensure that all users were viewing the same virtual environment, we implemented a client-server architecture utilizing Unity's networked multiplayer system (see Figure 3). We tracked each participant's physical movement and obtained 3D coordinate frames for all tracked objects (consisting of 2 controllers, 1 HMD, and 3 trackers). This enabled us to animate the self-avatars distinctly in the virtual world. The avatar's states and their location in the 3D coordinate space were submitted to the server, and synchronized to all remote clients. In each remote client, the corresponding avatars were animated based on these data. The state of each of the puzzle pieces were synchronized in a similar manner.

Aural communication was supported using Skype. In the course of experimentation, we identified that spatialized 3D audio as an important potential improvement to user engagement. This is an area for future study.

## 4. Experiment

The goal of the study was to extend the previous research and investigate whether the quality of the embodiment of users within avatars aids collaboration in SVE. To examine users' performance we manipulated the level of tracking and animation quality.

The experiment was approved by University College London Research Ethics Committee, project ID number 4547/009.

### 4.1. Hypotheses

- Hypothesis 1a (H1a): Solving the puzzle without a self-avatar will take longer time than with a self-avatar.
- Hypothesis 1b (H1b): Solving the puzzle with a self-avatar in the floating condition will take longer time than in the tracked feet condition
- Hypothesis 2a (H2a): Participants will collide more frequently with flowers without a self-avatar than with a self-avatar.
- Hypothesis 2b (H2b): Participants will detour farther around an obstacle in the floating feet condition than in the tracked feet condition. This will be measured by collision of the participant with a region around the flowers.

### 4.2. Methods

#### 4.2.1. Participants

Forty two participants, 21 dyads, were recruited from a University College London subject pool. 50% were male. We analyzed the ages and virtual reality experience for dyads in each condition. An ANOVA showed than there was no significant difference in age among the no avatar condition (*M* = 27.71, *SD* = 5.66), the floating feet condition (*M* = 26.86, *SD* = 7.47) and the tracked feet condition(*M* = 27.57, *SD* = 5.29). Using the Kruskal-Wallis H test, we found no significant difference in levels of virtual reality experience among the no avatar condition (*Median* = 3.5), the floating feet condition (*Median* = 4) and the tracked feet condition (*Median* = 4). All dyads were unacquainted with each other before the study. All participants were naive to the purposes of the study.

#### 4.2.2. Design

We chose a between subjects design to avoid possible carry-over effects. The main factor of the experiment was the avatar condition: no avatar vs. floating feet vs. tracked feet. Participants were randomly assigned to one of these body conditions.

#### 4.2.3. Procedure

Before beginning the experiment, participants at both sites were asked to fill out a brief demographic survey and a consent form. The experimenters in both laboratories gave the participants an overview of the jigsaw puzzle task that the participants would engage in. The experimenters outfitted the participants with one tracker on each foot, one around their waist, a Vive headset and a pair of Vive controllers. The experimenters calibrated matched-size self-avatars for them for the tracked feet and the floating feet conditions. The experimenters then guided the participants on how to manipulate objects using controllers. All participants wore all the trackers.

Once the participants had practised in the system, the experimenters set up an audio call via Skype and established the connection between two laboratories via the SVE. Participants were asked to solve jigsaw puzzles. After completing the puzzle, the experimenters at both laboratories terminated the connection, participants were asked to completed a questionnaire in private, and an informal post-experimental discussion then took place with the experimenters individually.

Finally, the participants were paid £5. The experiment took approximately 20 min.

#### 4.2.4. Data Collection

##### 4.2.4.1. Foot tracking and performance

The participant's head, waist, left foot and right foot movement were recorded every second throughout the experiment. The three coordinates (*x, y, z*) pertaining to each of these positions was output to a log file. These values provide a thorough representation of the participant's feet motion and along with a plan view of the environment, they allowed us to assess how participants may have been attempting to avoid obstacles.

Alongside the tracking data, the same log file also recorded participants' performance in terms of task completion time, corresponding to the time, in seconds, elapsed between the start of the puzzle session and the correct completion of the puzzle.

##### 4.2.4.2. Post-questionnaire

A post-experience questionnaire was then used to elicit information regarding the areas of interest discussed in the related works. The greatest part of the questionnaire is based on previous work (Slater et al., 2009; Yuan and Steed, 2010; Roth et al., 2017), since it has been shown to be a reliable indicator for ownership—the extent to which a participant perceived the virtual body to be themselves, agency—the sense of control of the virtual body, presence—a feeling of being in a place and co-presence—the sense of being with other people. Participants responded to a set of statements each with an associated 1–7 Likert scale, where an answer of 1 indicated complete disagreement, and 7 indicated complete agreement. The questionnaire, together with related result in the following analysis section, is presented in Table 1.

**Table 1:** Summary for the questionnaire responses (7-Likert scale).

**NO**.	**Questionnaire item**	**Kruskal-wallis H test**	***N* vs. *F***	***N* vs. *T***	***F* vs. *T***
Q1	During the experience I felt that the body I saw when looking down toward myself was my own body (even though it didn't look like me).	χ^2^(2) = 11.956, *p* = 0.003		*p* = 0.002	
Q8	During the experience I tried to avoid the virtual obstacle while performing the task.				
Q3	I felt like I controlled the virtual body as if it was part of my own body.	χ^2^(2) = 7.04, *p* = 0.03			
Q7	During the experience, I adjusted the movement of my real body according to the movement of the virtual body.	χ^2^(2) = 6.165, *p* = 0.046		*p* = 0.04	
Q10	During the experience I felt that the movements of the virtual body were my movements.	χ^2^(2) = 11.453, *p* = 0.003		*p* = 0.002	
Q4	There was a sense of being in the room which has office interiors.	χ^2^(2) = 6.175, *p* = 0.046	*p* = 0.04	
Q5	I think the virtual place is somewhere I visited, rather than just images I saw.				
Q6	There were times during the experience when the real world of the laboratory in which the experience was really taking place was forgotten.	χ^2^(2) = 7.062, *p* = 0.029	*p* = 0.049		
Q2	The experience was more like working with other people rather than interacting with a computer.				
Q9	There was a sense of being with the other people.				

*N stands for the no avatar condition, F is for floating feet, T is for tracked feet. Note: Only the significant P-value was reported*.

### 4.3. Results

#### 4.3.1. Task Completion Time

**Figure 4:**
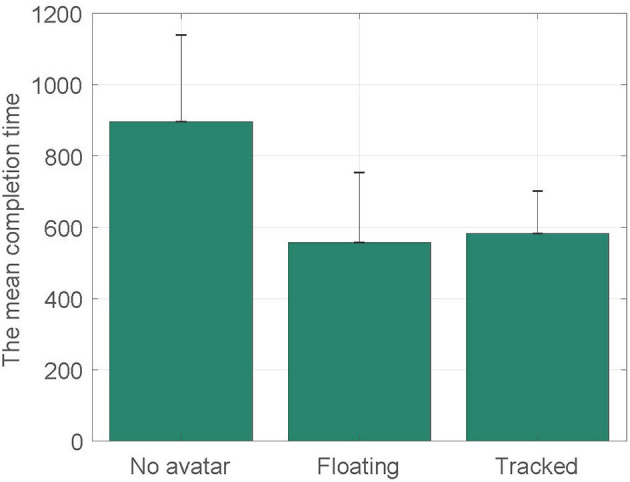
The mean task completion time for three conditions. Units of time are seconds.

Figure 4 presents the mean completion time for three body conditions. We can see that participants required longer time to solve the puzzle without a self-avatar (*M* = 895.56;*SD* = 224.3) than with a self-avatar. However, the means are very similar between the floating feet condition (*M* = 557.57;*SD* = 196.33) and tracked feet condition (*M* = 583.13;*SD* = 118.66).

A one-way ANOVA was conducted on the mean completion time, with three body conditions as between-subjects factors. There were no outliers, as assessed by boxplot; data was normally distributed for each group, as assessed by Shapiro-Wilk test (*p*>0.05); and there was homogeneity of variances, as assessed by Levene's test of homogeneity of variances (*p* = 0.128). The mean completion time was statistically significantly different between different conditions, *F*_(2, 20)_ = 6.623, *p* = 0.007. Tukey *post-hoc* analysis revealed that participants completed the task significantly slower in the no body condition than they did in the floating feet condition (*p* = 0.011) and the tracked feet condition (*p* = 0.019). H1a is supported. It also showed that there were no significant differences between the floating feet condition and the tracked feet condition (*p* = 0.967). H1b is not supported.

#### 4.3.2. Obstacle Avoidance Distance

**Figure 5:**
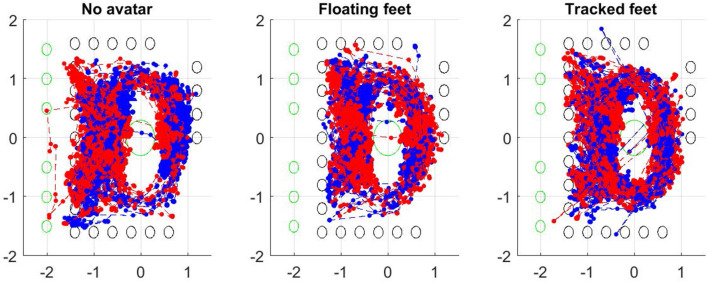
Paths taken from all participants in each condition. Left to right: no avatar, floating feet, tracked feet. Points represent left and right foot positions visualized every second. The green circles indicate locations of puzzle pieces, and the black circles location of flowers. Units are in meters.

Figure 5 shows the paths of all participants during the experiment when walking around to find each puzzle piece or standing still to complete the jigsaw, in each of the three conditions. Each point represents a left or right foot position. Denser areas on the plots indicate more steps on these areas. We performed a more detailed analysis of participants' obstacle avoidance behavior. For each dyad, the overall foot track points is the union of the sets of foot track points for left and right foot of both participants.

##### 4.3.2.1. Collision with the flowers

**Figure 6:**
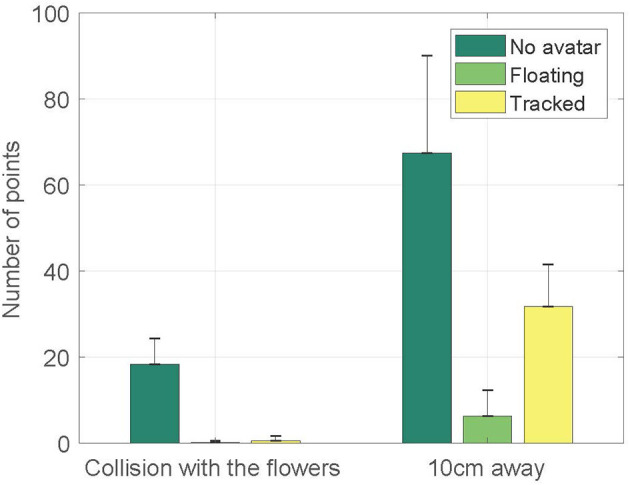
A classification of the paths of the participants for each condition. A point may collide with a flower (left-hand columns), or be with a 10 cm range of a flower (right hand columns). Each point would represents one second of time.

In the left-hand three columns, Figure 6 shows the number of time points at which the avatar in each condition was standing on the flowers (i.e., one of the foot track points was inside the bounding circle of the flower). Note this only roughly approximately to time inside the target because points are sampled every second.

A one-way Welch ANOVA was conducted to determine if number of points was different for no avatar, floating feet and tracked feet conditions. There were no outliers and the data was normally distributed for each conditions, as assessed by boxplot and Shapiro-Wilk test (*p*>0.05), respectively. Homogeneity of variances was violated, as assessed by Levene's Test of Homogeneity of Variance (*p* = 0.01), hence the use of the Welch ANOVA. The number of points was statistically significantly different between different conditions, Welch's *F*_(2, 8.819)_ = 29.466, *p* < 0.0005. Games-Howell *post-hoc* analysis revealed that the number of points in the no avatar condition was significantly higher than the floating feet condition (*p* = 0.001) and the tracked feet condition (*p* < 0.0005). H2a is supported. It also shows that there was no significant difference between the floating feet condition and the tracked feet condition (*p* = 0.629).

##### 4.3.2.2. Proximity to the flowers

In the right hand three columns, Figure 6 shows the number of time points at which the avatar in each condition was proximate to the flowers (i.e., one of foot track points was within 10 cm of the bounding circle of the flower).

A one-way Welch ANOVA was conducted to determine if number of points was different for no avatar, floating feet and tracked feet conditions. There were no outliers and the data was normally distributed for each conditions, as assessed by boxplot and Shapiro-Wilk test (*p*>0.05), respectively. Homogeneity of variances was violated, as assessed by Levene's Test of Homogeneity of Variance (*p* = 0.001), hence the user of the Welch ANOVA. The number of points was statistically significantly different between different conditions, Welch's *F*_(2, 10.308)_ = 33.808, *p* < 0.0005. Games-Howell *post-hoc* analysis revealed that the number of points in the no avatar condition was significantly higher than the floating feet condition (*p* = 0.001) and the tracked feet condition(*p* = 0.12). It also showed that the number of points in the tracked feet condition was significantly higher than the floating feet condition (*p* < 0.0005). H2b is supported.

#### 4.3.3. Post-questionnaire

**Figure 7:**
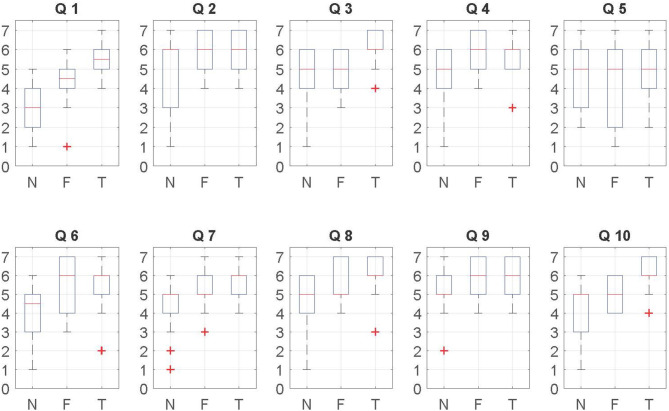
Box-plots for questionnaire items associated with Table 1. Medians, interquartile ranges, and full ranges are shown. N stands for the no avatar condition, F is for floating feet, T is for tracked feet.

Figure 7 shows an overview of the data. We created an aggregate response by calculating the mean ranks of the responses to each questionnaire item given by the participants from both laboratories. The responses was analyzed using the Kruskal-Wallis H test. If the Kruskal-Wallis H test was statistically significant, pairwise comparisons were performed using Dunn's (1964) procedure with a Bonferroni correction for multiple comparisons. Overall, we found some (Q1, Q3, Q4, Q6, Q7, and Q10) significant effects due to the self-avatars. However, no significant effects were found between the floating feet and tracked feet conditions directly, though there are indirect effects where only one of floating feet or tracked feet is different from no avatar (see below). Table 1 provides the summary of the results. Adjusted *p*-values are presented, and only significant results are shown.

Regarding the ownership-related questions (Q1, Q8 in Figure 7 and Table 1), we observe that participants with a self-avatar tended to give higher subjective ratings than those without self-avatar. We also found slightly higher ratings for the tracked feet condition in terms of the virtual hand being part of their body. According to the agency results (Q3, Q7, and Q10 in Figure 7 and Table 1), we observe that participants elicited a slightly stronger sense of agency with the foot tracking than without the foot tracking. Statistically significant results were found in Q1, Q7, and Q10, but not Q3 and Q8.

According to the presence (Q4, Q5, and Q6) and co-presence results (Q2 and Q9) in Figure 7 and Table 1. No effects are found for Q5. Thus the presence question that drew out a distinction were Q4 and Q6. The results suggest that subjective rating of presence is enhanced by providing self-avatars. This is similar to the previous work (Slater et al., 1995; Steed et al., 1999). No main effects were found for copresence for any question.

#### 4.3.4. Observations

We analyzed transcripts from our interviews and video footage, focusing on how participants collaborated (see **Figure 10**).

##### 4.3.4.1. Ownership

In the avatar conditions, all participants noted the flowers and followed a clear path to pick up the pieces, although they considered that the virtual feet were able to go through virtual obstacles. Surprisingly, in the no avatar condition, two participants did not notice the flowers while solving puzzles. One of them commented (when asked “did you notice the flowers?”)

“*No, I didn't. I was concentrated on solving puzzles.”*

##### 4.3.4.2. Agency

Our post interview further supports the findings of the post-questionnaire. One participant in the floating feet condition directly commented (when asked “Did you find it easy to control your avatar?”)

“*I can control the movements of arms; but the movements of the legs are not precise as the movements of arm.”*

Another commented (when asked “did you notice the flowers?”)

“*I am aware of the patch of flowers that's just in front of me while picking up the jigsaw piece. I didn't mess with those flowers, but sometimes I find it hard to move my foot onto an exact location.”*

By contrast, in the words of one participant in the tracked feet condition.

“*I carefully navigated around the flowers. I think I didn't step on a single flower by accident.”*

This might also indicate that participants felt greater confidence in their ability to control the movement of legs.

##### 4.3.4.3. Other communicative actions

**Figure 8:**
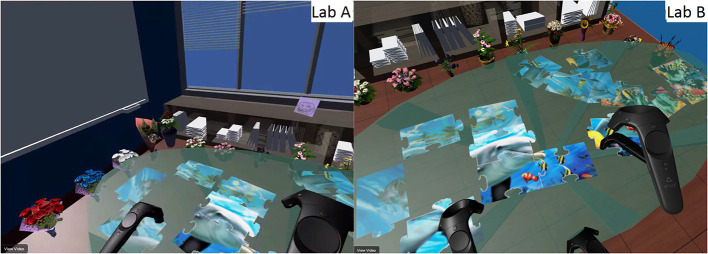
Users accessing the same puzzle piece in no avatar condition. Each pair of screen shots was simultaneously captured from the first-person view of each user within the dyad in the succeeding Figures.

In both self-avatar condition, we saw that the avatar might more easily convey a sense of on-going activity. For example, the limbs can more accurately indicate which puzzle piece a given user is going to access. However, due to the lack of visibility in the no self-avatar condition, we observed quite a few cases where two participants tried to access the same puzzle piece (see Figure 8).

One participant commented:

“*He often tried to take control of my pieces.”*

Thus, this introduces a possibility of interference and confusion, where one participant's actions potentially disturb the productivity of others.

**Figure 9:**
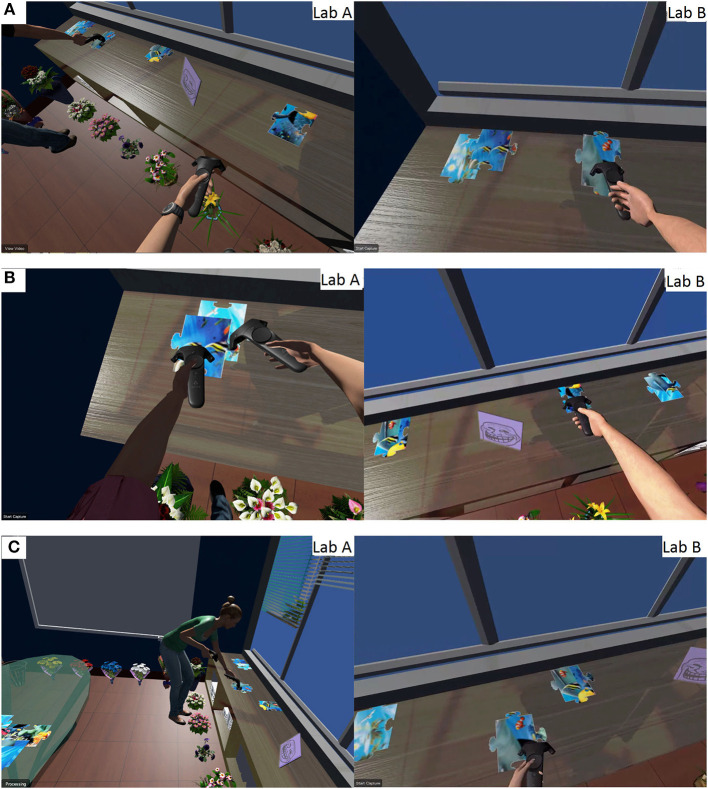
Participants with tracked feet, as opposed to floating feet, placed their feet closer to obstacles. **(A)** Floating feet. **(B)** Tracked feet. **(C)** Tracked feet.

We observed participants taking a clear path to collect pieces from some shelves through the narrow gap between two flowers in the tracked feet condition (see Figure 9).

**Figure 10:**
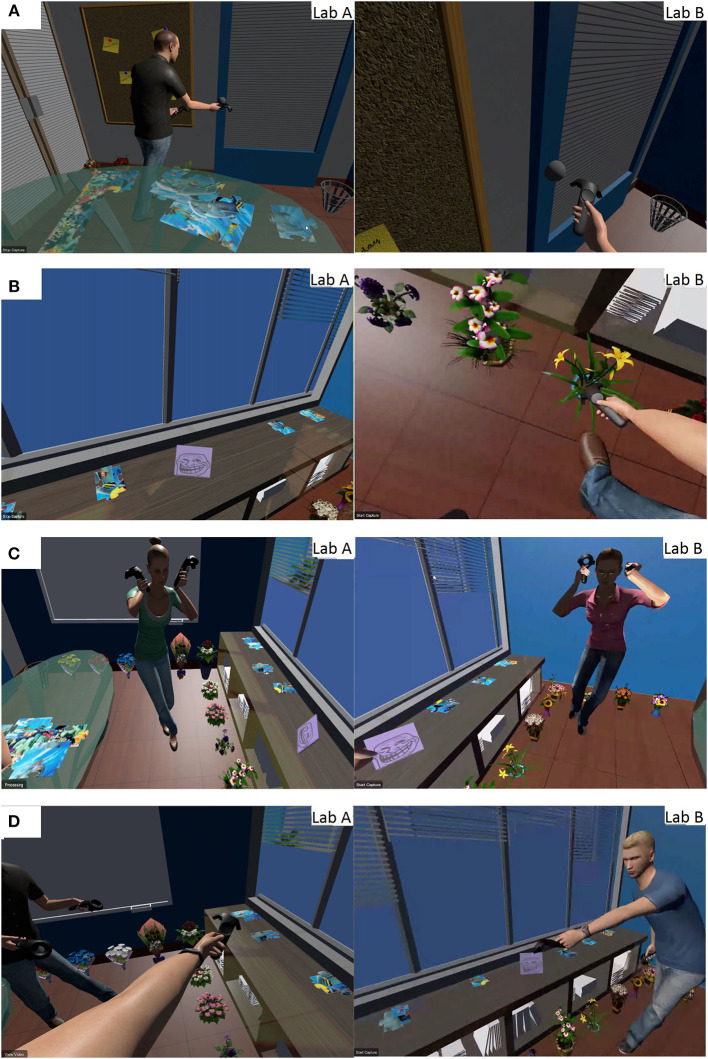
Various communicative acts for different conditions. **(A)** Exploring the virtual office in the floating feet condition. **(B)** Exploring the virtual office in the tracked feet condition. **(C)** One user making a cheerleading pose and the other mimicking her pose. **(D)** One participant pointing and the other just agreeing.

We also observed the participants making several other communicative actions such as looking at each other (see Figure 10), nodding, shrugging and waving good-bye. One of the most interesting actions was that one participant spontaneously made a cheerleading pose, and the other mimicked her pose (see Figure 10). In both avatar conditions, some groups maintained a conversation during the experiment, constantly updating each other on the visible portions of the pieces, guesses of what the complete picture might be, and strategies for collaboration. Some groups didn't feel a need to constantly communicate with their partner verbally, as a quick glance was sufficient. We frequently observed deictic references such as “this” or “that” while pointing (see Figure 10). In contrast, participants in the no avatar condition often gave detailed instructions and described specific pieces to ensure the other partner could clearly understand their advice.

## 5. Discussion

### 5.1. Main Results

The results on the task completion time support H1a. H1b is not supported, but this is not surprising in retrospect. Given that the arms were most relevant to the task, it is reasonable to find larger differences between the no avatar condition and the two avatar conditions, than between the floating feet and tracked feet conditions.

The results of the obstacle avoidance behavior and movement analysis support both H2a and H2b. We sampled foot positions every second. We compared collision with the flowers and proximity to the flowers among no avatar, floating feet and tracked feet conditions. Statistically significant results were found.

The results of questionnaires indicated that the floating feet condition reduces the score on the ownership and agency questions compared to tracked feet condition. Also, the self-avatar condition increases the score on ownership, agency and presence question compared to no self-avatar condition.

### 5.2. Limitations and Future Work

In designing a controlled experiment we had to make several choices about the situation of the user, the tasks provided and the representation of the self-avatar for the user. For example, each of the male and female avatars we used was calibrated based on the participant's height. This would mean that the length of the arm could be subtly wrong. As the result, avatar's wrist is seldomly twisted, and the controller occasionally moved out of the hand because the arms were too short to extend to meet it. Any or all of additional calibration, matching the body shape of the user and tracking the body and fingers more accurately might improve the user experience.

Our SVE system used generic avatars for the sake of generality and simplicity. However, several studies have reported the impact of the degree of individualization and personalization of users' avatars as well as the impact of the degree of immersion on typical psychophysical factors in VE (e.g., Jung et al., 2018; Waltemate et al., 2018). We plan to compare personalized avatars and generic avatars by investigating the impact of agency and body ownership on trust formation.

In this initial study, our measures of the behavioral impact of foot tracking are based on measures of collision with the flowers. This simple measure did identify a difference in participants' behavior between floating and tracked feet. To dig deeper, we plan to analyze the average stride length or speed of movement, curvature of paths to estimate some measure of naturalness. We also plan to evaluate whether users looked at their feet or perhaps even gestured with their feet. Finally, we can imagine tasks that specifically solicit interactions with the feet. We expect that there are more subtle differences to be found between floating and tracked feet.

## 6. Conclusion

We implemented an SVE system where each user uses the commercially available HTC Vive headset and controllers combined with three Vive trackers. Users in separate physical spaces had the opportunity to embody an avatar and interact in an SVE. As consumer virtual reality equipment has become cheaply available, the low cost and ease of setup make this an interesting platform for next generation telecollaboration.

In an experiment, we examined what level of tracking detail is required in order to successfully embody users in collaborative situations. Results demonstrated that users with self-avatars completed the task more quickly that users without self-avatar. The addition of foot tracking had no significant effect in term of the task performance time. However, a deeper look at the participants' behavior in proximity to objects showed some differences. We found that more collisions with obstacles occurred in the no avatar condition than in either avatar conditions. Furthermore, we found that participants stood farther from and walked farther around obstacles in the floating feet condition than in the tracked feet condition. We observed various interesting acts such as, participants carefully placing their feet between flowers while picking up the puzzle piece in the tracked feet condition. Lastly, users' self-reports also supported the importance of foot tracking in letting users tread carefully around obstacles.

Our results have important implications for the design of VR HMD systems: simply adding a self-avatar could provide better remote collaboration experience and enabling foot tracking can alter users' obstacle avoidance behavior. Not only does being able to see where other participants stand provide enhanced plausibility to the environment, but it opens up whole new types of interaction.

## Ethics Statement

The study was approved by University College London Research Ethics Committee, project ID number 4547/009.

## Author Contributions

YP wrote the code, ran the study, analyzed the data, and wrote the paper. AS posed the original hypothesis and supervised YP through each stage of the work including: writing the code, running the study, analyzing the data, and writing the paper.

### Conflict of Interest

The authors declare that the research was conducted in the absence of any commercial or financial relationships that could be construed as a potential conflict of interest.
